# A retrospective survey of patients with hereditary transthyretin-mediated (hATTR) amyloidosis treated with patisiran in real-world clinical practice in Belgium

**DOI:** 10.1007/s13760-023-02188-z

**Published:** 2023-02-24

**Authors:** Jan L. De Bleecker, Kristl G. Claeys, Stéphanie Delstanche, Vinciane Van Parys, Jonathan Baets, Sébastien Tilleux, Gauthier Remiche

**Affiliations:** 1 Department of Neurology, University Hospital Ghent, Ghent, Belgium; 2 Department of Neurology, University Hospitals Leuven, Laboratory for Muscle Diseases and Neuropathies, KU Leuven, Leuven, Belgium; 3 Department of Neurology, Centre Hospitalier Universitaire de Liège, Liège, Belgium; 4 Department of Neurology, Cliniques Universitaires Saint-Luc, Brussels, Belgium; 5 Department of Neurology, Antwerp University Hospital, Faculty of Medicine and Health Sciences, Translational Neurosciences, UAntwerpen, Antwerp, Belgium; 6 Alnylam Belgium BVBA, Schumanplein 6, 1040 Brussels, Belgium; 7 Department of Neurology, Hôpital Erasme, Université Libre de Bruxelles (ULB), Brussels, Belgium

**Keywords:** Patisiran, hATTR, Real-world evidence, Polyneuropathy

## Abstract

**Introduction:**

Hereditary transthyretin-mediated (hATTR) amyloidosis, a genetic disease caused by mutations in the transthyretin gene, leads to progressive sensory and autonomic neuropathy and/or cardiomyopathy and is associated with renal and ophthalmologic manifestations and a poor prognosis.

**Methods:**

This is a retrospective study based on data collected from the medical records of patients with hATTR amyloidosis treated with patisiran between 01 July 2018 and 01 February 2021. Six Belgian neuromuscular reference centers participated, covering all patisiran-treated hATTR amyloidosis patients at the study time. This study was conducted to collect data requested in the context of the reimbursement of patisiran in Belgium.

**Results:**

Thirty-one patients were diagnosed with hATTR amyloidosis with polyneuropathy, Coutinho stage 1 or 2, and eligible for active treatment during the data collection period. Of the hATTR amyloidosis patients treated with patisiran (*n* = 12), seven and five had polyneuropathy stages 1 and 2, respectively. Six patients had cardiac symptoms (New York Heart Association class 2 or above). Follow-up information was available for nine patients. Following patisiran treatment, eight patients showed stable or improved assessments for most neurological or cardiological parameters. Only one patient presented with worsening statuses at the end of the data collection period.

**Conclusions:**

The patients with hATTR amyloidosis in Belgium have similar baseline demographics and disease characteristics to those studied in the patisiran APOLLO study and show a similar therapeutic response in the real-world, altering the expected disease progression in most patients.

**Supplementary Information:**

The online version contains supplementary material available at 10.1007/s13760-023-02188-z.

## Introduction

Hereditary transthyretin-mediated (hATTR) amyloidosis is a rare (prevalence: 1/1,000,000 persons [1]), potentially fatal, autosomal dominant, multisystemic disease caused by mutations in the transthyretin (*TTR*) gene with as main features a progressive sensory and autonomic motor neuropathy and/or a cardiomyopathy [2–5]. Besides peripheral nerve disease or cardiac manifestations, patients may also present with central nervous system, ocular, and renal manifestations [1, 6]. hATTR amyloidosis is caused by the deposition of misfolded precursor protein TTR as insoluble amyloid fibrils in multiple organs, ultimately disrupting normal tissue structure and function [7]. hATTR amyloidosis with polyneuropathy has sensory and motor impact. Left untreated, patients will eventually develop severe functional limitations caused by sensory abnormalities and motor nerve involvement, resulting in pain, weakness, and gait disturbance. Autonomic dysfunction causes debilitating gastrointestinal and cardiovascular symptoms. This progressive morbidity results in severe disability and cachexia, leaving patients unable to perform daily activities and making them dependent on their surroundings, thereby also affecting their mental health [2]. Death usually results from heart failure with a median survival without treatment of about 10 years for patients with polyneuropathy only [8–11]. Patients with predominant cardiomyopathy have a median survival of 3.4 years, with the common cause of death being progressive heart failure or cardiac arrhythmia [12–14].

In terms of symptoms, four stages of hATTR amyloidosis with polyneuropathy (Coutinho Familial Amyloid Polyneuropathy (FAP) stages) are distinguished [15]. Patients with stage 0 disease are asymptomatic, patients with stage 1 (mild) disease are ambulatory, patients with stage 2 (moderate) disease are ambulatory but require assistance and/or have involvement of the upper limbs, and patients with stage 3 (severe) disease are bedridden or wheelchair-bound [16].

Orthotopic liver transplantation can be a treatment option for patients in an early stage of the disease without a heart condition but is associated with high morbidity and is limited by the lack of available organs [17]. Recently, effective therapies have emerged outside of orthotopic liver transplant that have revolutionized the management of hATTR amyloidosis [18]. There are currently two drug treatments available in Belgium for adult hATTR amyloidosis patients with polyneuropathy in stage 1 or 2: tafamidis and patisiran. Inotersen is a third therapeutic option approved by EMA, but not commercialized in Belgium. All treatments, including orthotopic liver transplantation, aim at reducing the amount of circulating amyloidogenic protein. Tafamidis, a TTR tetramer stabilizer approved to treat adult hATTR patients with stage 1 polyneuropathy [19], is widely used for patients with hereditary or wild-type ATTR with cardiomyopathy.

Patisiran is an RNAi therapeutic, which contains a small interfering RNA formulated as lipid nanoparticles to deliver to hepatocytes, the primary source of TTR protein in the circulation. It targets variant and wild-type TTR messenger RNA (mRNA), resulting in a reduction in serum TTR. It is the only available and reimbursed treatment in Belgium targeting the underlying cause of the disease by reducing the production of liver-derived circulating amyloidogenic TTR [4, 20–22]. In the phase 3 APOLLO Study (a randomized, double-blind, placebo-controlled clinical trial), patisiran showed significant improvement in polyneuropathy and all secondary endpoints compared with placebo at 18 months, in patients with hATTR amyloidosis with polyneuropathy [20]. Noteworthy, exploratory endpoints have shown significant improvement in cardiac structure and function. These data show promising trends in the treatment of cardiac amyloidosis and will require additional clinical trials to confirm these findings [21]. Patisiran 0.3 mg/kg every 3 weeks (Q3W) is now approved in more than 30 countries for the treatment of hATTR amyloidosis in adult patients with polyneuropathy [23, 24]. A new RNAi therapeutic, vutrisiran, showed promising results in clinical trials, but is not available yet in Belgium [25].

Inotersen is an antisense oligonucleotide to the TTR 3’-untranslated mRNA inhibiting hepatic TTR production [26]. It decreases mutated and wild-type TTR levels, leading to a reduced formation of TTR amyloid fibril deposits, slowing or halting disease progression.

We conducted a retrospective study to gather information about the epidemiology of adult hATTR amyloidosis patients with polyneuropathy stage 1 or 2 in Belgium, its clinical practice in terms of treatment, the real-world use of patisiran, and the clinical outcomes associated with patisiran treatment. The main objectives of the study included: (1) determination of the current number of hATTR amyloidosis patients in Belgium, including an understanding of clinical practice in terms of treatments as of 01 February 2021, and (2) determination of the clinical impact of patisiran use in adult patients with hATTR amyloidosis in a real-world setting in terms of neurological and cardiac symptoms for the period starting from 01 July 2018 to 01 February 2021.

## Methods

This study was conducted to collect data requested in the context of a convention between Alnylam Belgium and the Belgian Health Insurance Agency (RIZIV/INAMI) for the reimbursement of patisiran in Belgium.

### Study design and participants

This study is a retrospective study based on data from the medical records of patients with hATTR amyloidosis treated with patisiran from 01 July 2018 to 01 February 2021. Six out of seven Belgian neuromuscular reference centers (NMRCs) participated in the study. These six centers include all centers with hATTR amyloidosis patients under treatment at the study time. 

The study population included adult patients (≥ 18 years old) with hATTR amyloidosis confirmed by genetic testing, with polyneuropathy stage 1 or stage 2. This study did not include asymptomatic (stage 0) or stage 3 patients or hATTR amyloidosis patients with only cardiomyopathy (i.e., no polyneuropathy).

**Fig. 1:**
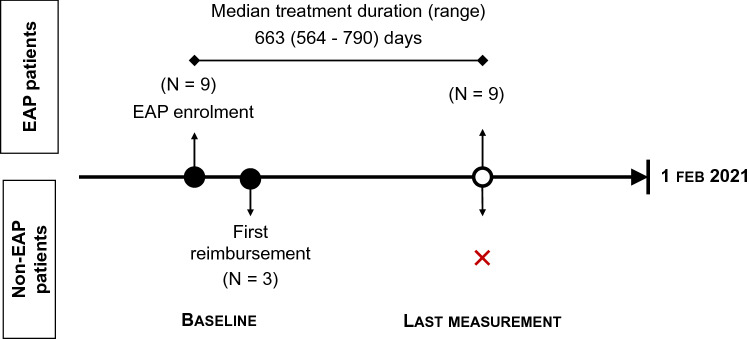
Overview of available data/median treatment duration in the Belgian patisiran population included in the RWE study

Nine patients started treatment with patisiran during an Expanded Access Program (EAP) (Fig. 1). Three patients started treatment with patisiran after reimbursement was granted (01 December 2019). As those three patients have been on treatment for less than 12 months, no follow-up data were available. Patisiran was administered as an 80-min intravenous infusion at a 300 µg/kg dose every 3 weeks. All patients received premedication (including corticosteroids, H1 and H2 blockers, and paracetamol) to reduce the risk of infusion-related reactions. Vitamin A supplementation at approximately 2500 IU vitamin A per day is advised for patients treated with patisiran.

This study was a retrospective data survey without patient enrolment. As per Belgian law, surveys fall outside clinical trial legislation (07 May 2004) and, therefore, do not require approval by a medical ethics committee (EC) or an Institutional Review Board. The study protocol and informed consent (or the approval for exemption from consent) were provided to the participating centers' ethics committees. In the context of a scientific survey, a waiver for informed consent was required by the medical ECs of all participating centers, as only aggregated data were returned for analysis. In addition, the medical ECs of two participating centers (UZ Leuven and UZ Gent) specifically requested the submission of an informed consent form for approval. All patients signed informed consent. The study was conducted per the declaration of Helsinki. All patient data were anonymized, and data privacy was contained during the investigation.

The study was sponsored by Alnylam and performed according to the study protocol and Modis Life Science’s standard operating procedures. Patient confidentiality was ensured following the study agreement between Modis Life Sciences and the participating sites. The detailed study protocol can be consulted in supplementary materials (SI).

### Data collection

Data recorded in patient’s medical records from 01 July 2018 to 01 February 2021was collected by qualified site personnel using a data collection form developed by Modis Life Sciences. This study is an observational survey and uses only data collected in routine clinical practice.

Only data available at the time of the survey were recorded. Hence, no substitution, imputation, or other correction methods were used to complete missing values.

### Neurological and cardiological parameters measured throughout the study

Neurological parameters were measured by the Polyneuropathy Disability score (PND score), the Neuropathy Impairment Scale–Lower Limbs (NIS-LL), an electromyogram (EMG), the Functional Independence Measurements (FIM) score, ACTIVLIM (the 3-level scale (impossible/difficult/easy) or the 36-points scale) and/or the Compound Autonomic Dysfunction (CADT) score. In addition, orthostatic hypotension was measured in many patients during consultation; however, the values were not systematically collected. Assessment of orthostatic hypotension was thus based physician clinical judgement from history data (symptoms) and confirmatory measurements when judged appropriate.

Cardiological symptoms were assessed with the NYHA score, an electrocardiogram (ECG), and echocardiography measuring the left ventricular ejection fraction (LVEF).

### Statistical analyses

The statistical analysis of the collected information was descriptive and consisted of frequencies for categorical variables and means, standard deviations, medians, and ranges for continuous variables. No comparative statistical analysis was performed, as the small population size would yield results with limited relevance. Therefore, the results consist of a descriptive presentation of the collected data.

## Results

### Patient characteristics

At data cut-off (01 February 2021), 31 patients were diagnosed and followed up for hATTR amyloidosis with polyneuropathy stage 1 or 2 in Belgium. Of these, 4 patients underwent a liver transplant, 22 were under active treatment (with either tafamidis or patisiran), and 5 symptomatic patients were not under active treatment (patient preference not to be treated or reimbursement file under review in recently diagnosed patients). It is possible that some of these five patients have recently been diagnosed and could benefit from treatment in the future. A total of 12 patients were treated with patisiran over the study period.

Table 1 presents the baseline characteristics for continuous and categorical values for all 12 patients (both EAP and non-EAP patients) prior to treatment. The median age was 63 years (range 32–81 years). There was an even distribution between patients with polyneuropathy FAP stage 1 (*n* = 7) and stage 2 (*n* = 5). Most patients treated with patisiran were treatment naïve (10 out of 12 patients). In addition, two patients had previously been treated with tafamidis, of which one patient also had a heart transplant. The multisystemic nature of the disease is illustrated by the presence of neurological and/or cardiological symptoms evidenced by different scales and measurements.

**Table 1:** The baseline characteristics for continuous and categorical values for all 12 patients (both EAP patients and non-EAP patients) prior to treatment

Baseline Characteristics	EAP patients (*N* = 9)	Non-EAP patients (*N* = 3)	Total (EAP and non-EAP patients) *N* = 12
Age (median, range), years	–	–	63 (32–81)
Weight (median, range), kg	82.0 (45.0–110.0)	66.0 (65.4–70.0)	73.7 (45.0–110.0)

Neurological parameters			
FAP (*n*, %)	*N* = 9	*N* = 3	*N* = 12
Stage 1	6 (67)	1 (33)	7 (58)
Stage 2	3 (33)	2 (67)	5 (42)
PND (*n*, %)	*N* = 7	*N* = 2	*N* = 9
1	4 (44)	–	4 (33)
2	2 (22)	–	2 (17)
3a	1 (11)	1 (33)	2 (17)
3b	0 (0)	1 (33)	1 (8)
NIS-LL	*N* = 5	*N* = 2	*N* = 7
Median (range)	31.0 (6–58)	30 (3–56)	31.0 (3–58)
Mean (SD)	27.0 (21.42)	29.5 (37.48)	27.7 (23.16)
EMG (*n*, %)*	*N* = 9	*N* = 3	*N* = 12
Normal	1 (11)	–	1 (8)
abnormal	8 (89)	3 (100)	11 (92)
FIM Score	*N* = 9	*N* = 2	*N* = 11
Median (range)	121.0 (92–126)	98 (85–110)	115.0 (85–126)
Mean (SD)	116.3 (11.0)	97.5 (17.68)	112.9 (13.64)
ACTIVLIM (*n*, %) 3 point scale (easy/difficult/impossible)	*N* = 5	*N* = 2	*N* = 7
Easy	(1, 11)	−	(1, 8)
Difficult	(3,33)	(1, 33)	(4, 33)
Other scale (36 point scale) Median (*n*, %)	34 (1, 11)	26 (1, 33)	30 (2, 16)
CADT	*N* = 3	*N* = 2	*N* = 5
Median (range)	15.0 (9–18)	12 (11–13)	13.0 (9–18)
Mean (SD)	14.0 (4.58)	12.0 (1.41)	13.2 (3.49)
Orthostatic hypotension	*N* = 9	*N* = 3	*N* = 12
Yes (*n*, %)	4 (44)	2 (67)	6 (50)
No (*n*, %)	5 (56)	1 (33)	6 (50)

Cardiological parameters			
NYHA score (*n*, %)	*N* = 9	*N* = 2	*N* = 11
1	5 (56)		5 (42)
2	3 (33)	2 (67)	5 (42)
3	1 (33)		1 (8)
ECG* (*n*, %)	*N* = 9	*N* = 3	*N* = 12
Normal	5 (56)	1 (33)	6 (50)
Abnormal	4 (44)	2 (67)	6 (50)
Echocardiography–LVEF	*N* = 9	*N* = 3	*N* = 12
Median (range)	60.0 (40–61)	50.0 (45–78)	–
Mean (SD)	54.0 (9.57)	57.7 (17.84)	–
Normal (*n*, %)	6 (67)	–	6 (50)
Abnormal (*n*, %)	3 (33)	3 (100)	6 (50)

*CADT* compound autonomic dysfunction, *EAP* early access program, *ECG* electrocardiogram, *EMG* electromyogram, *FAP* familial amyloid polyneuropathy, *FIM* functional independence measurements, *LVEF* left ventricular ejection fraction, *NIS* neuropathy impairment score, *NYHA* New York heart association, *PND* Polyneuropathy Disability

^*^Based on physician assessment

For the PND score, four patients had a score of 1, two patients each had a score of 2 and 3a, respectively, and one patient had a PND score of 3b. A median NIS-LL score of 31.0 was reported for seven patients. An abnormal EMG was recorded for 11 patients. A median FIM score of 115.0 was reported for 11 patients. ACTIVLIM results were available for seven patients. The ACTIVLIM data collected using the 36-points scale showed one patient each with a score of 26 and 34, respectively. Using the three-level scale (impossible/difficult/easy), one patient rated the required activities as easy, while four patients rated the required activities as difficult. Orthostatic hypotension occurred in half of the patients (*n* = 6).

About half of the patients (*n* = 6) had cardiac symptoms with a NYHA score of 2 or above: five patients had slight limitation of physical activity (NYHA 2), and one patient had marked limitation of physical activity (NYHA 3). Six patients had abnormal ECG and LVEF findings.

### Real-world treatment use of patisiran

The mean and median treatment duration for the 12 patisiran-treated patients included were 551.1 days and 700 days (range: 35–815 days). Patients received a median and mean of 25.5 and 32 treatment cycles (range: 2–39). The mean and median number of missed cycles were 0.3 and 0 (range 0–1). Temporary discontinuations were recorded for four patients, corresponding to one dose missed for each discontinuation. The single permanently discontinued patient from patisiran treatment had died from heart failure. Overall, 98.9% of the planned treatment cycles were administered over the total study period.

### ***Impact of patisiran on neurological parameters (****Table **2****)***

**Table 2:** Overview of neurological and cardiological parameters at baseline and at the last measurement compared of patients who started treatment with patisiran during the EAP (*N* = 9)

Baseline		Last measurement	
Neurological parameters		Neurological parameters	
FAP –*n* (%)	*N* = 9	FAP–*n* (%)	*N* = 9
1	6 (67)	Improved (vs baseline)	0 (0)
2	3 (33)	Stable (vs baseline)	8 (89)
		Worsened (vs baseline)	1 (11)
PND –*n* (%)	*N* = 7	PND –*n* (%)	*N* = 4
1	4 (44)	Improved (vs baseline)	1 (11)
2	2 (22)	Stable (vs baseline)	3 (33)
3a	1 (11)	Worsened (vs baseline)	0 (0)
3b	0 (0)		
NIS-LL –*n* (%)	*N* = 5	NIS-LL–*n* (%)	*N* = 4
Median/mean NIS-LL–scale (min/max)	31.0/27.0 (6/58)	Median/mean difference in NIS-LL scale vs baseline (min/max)	− 1/− 1.5 (− 5/0)
		Improved (vs baseline)	2 (22)
		Stable (vs baseline)	2 (22)
		Worsened (vs baseline)	0 (0)
EMG* –*n* (%)	*N* = 9	EMG* –*n* (%)	*N* = 9
Normal	1 (11)	Improved or stable (vs baseline)	9 (100)
Abnormal	8 (89)	Worsened (vs baseline)	0 (0)
FIM score	*N* = 9	FIM score	*N* = 9
Median/mean FIM score (min/max)	121.0/116.3 (92/126)	Median/mean difference in FIM score vs baseline (min/max)	0.0/0.8 (− 8/14)
		Improved (vs baseline)	2 (22)
		Stable (vs baseline)	6 (67)
		Worsened (vs baseline)	1 (11)
Orthostatic hypotension* –*n* (%)	*N* = 9	Orthostatic hypotension*–*n* (%)	*N* = 9
Yes	4 (44)	Improved (vs baseline)	2 (22)
No	5 (56)	Stable (vs baseline)	7 (78)
		Worsened (vs baseline)	0 (0)

Cardiologic parameters		Cardiologic parameters	
NYHA score –*n* (%)	*N* = 9	NYHA score–*n* (%)	*N* = 9
1	5 (56)	Improved (vs baseline)	1 (11)
2	3 (33)	Stable (vs baseline)	7 (78)
3	1 (11)	Worsened (vs baseline)	1 (11)
ECG* –*n* (%)	*N* = 9	ECG*–*n* (%)	*N* = 9
Normal	5 (56)	Improved (vs baseline)	0 (0)
Abnormal	4 (44)	Stable (vs baseline)	9 (100)
		Worsened (vs baseline)	0 (0)
Echocardiography–Median/mean % LVEF (min/max)	60.0/54.0 (40/61) (*N* = 9)	Echocardiography–Median/mean difference in % LVEF (min/max)	5.0/9.5 (− 5/37) (*N* = 9)
Normal	6 (67)	Improved (vs baseline)	0 (0)
Abnormal	3 (33)	Stable (vs baseline)	9 (100)
		Worsened (vs baseline)	0 (0)

*EAP* expanded access program, *ECG* electrocardiogram, *FAP* familial amyloid polyneuropathy, *FIM* functional independence measurements, *LVEF* left ventricular ejection fraction, *NIS-LL* neuropathy impairment score in lower limbs, *NYHA* New York heart association, *PND* polyneuropathy disability

^*^Based on physician assessment

Follow-up data (Fig. 1) were only available for patients in the EAP (9 patients), as these patients had more prolonged treatment exposure.

Most patients (*n* = 8) reported stable polyneuropathy with an unchanged FAP stage at the last time point compared to baseline (Table 2). Only one of the nine patients presented a worsening in the FAP stage recorded at the end of the data collection period. The PND score and the NIS score were only reported for four patients at the last measurement. One patient reported improvement in PND score, and three reported stabilization. The NIS score was stable in two patients and improved in two patients. Compared to baseline, the FIM score remained stable for six patients, improved for two patients, and worsened in one patient. Fewer patients reported orthostatic hypotension after the start of the treatment (*n* = 2). At the last measurement, seven patients reported a stable status and two an improved status. EMG data improved or remained stable compared to baseline in all nine patients. No conclusions can be made for the ACTIVLIM score, and the CADT score, as insufficient data were available at the last measurement.

### ***Impact of patisiran on cardiological parameters (****Table **2****)***

Follow-up data (Fig. 1) were only available for patients in the EAP (9 patients), as these patients had more prolonged treatment exposure.

The NYHA score improved in one patient, was stable for seven patients, and worsened in one patient. ECG readings and the echocardiography status (LVEF) from all patients were stable at the last measurement. The mean LVEF increased over time from 57.5% at baseline to 63.5% at the last measurement. The mean increase in LVEF was 9.5% at the last measurement compared to baseline.

## Discussion

This retrospective study was conducted to gather information about the epidemiology of adult hATTR amyloidosis patients with polyneuropathy, stage 1 or 2, in Belgium and its clinical practice in terms of treatment use and the real-world use of patisiran and the clinical outcomes associated with it. As reported in this study, the evaluation of different neurological and cardiological parameters is in line with recent recommendations for a multisystem approach to best capture the course of the disease [27]. The study shows that the efficacy of patisiran, as reported in the initial pivotal clinical trials [4, 20, 21], is confirmed in real-world clinical practice over a median period of 663 days (564–790) since treatment initiation.

In Belgium, 31 patients with hATTR amyloidosis with polyneuropathy stage 1 or 2 were followed in the NMRCs who had hATTR amyloidosis patients under treatment at the time of the study who participated in this study (6 out of 7 expert centers). Considering all NMRCs, this would result in an estimated prevalence of 2.68 per million persons in Belgium. This estimated prevalence is consistent with the non-endemic prevalence observed in EU countries [28, 29].

The patients with hATTR amyloidosis polyneuropathy in Belgium have similar baseline demographics and disease characteristics to those studied in the pivotal patisiran APOLLO study [20]. Patients are evenly distributed between polyneuropathy stages 1 and 2. About half of the patients had cardiac symptoms, with a NYHA score of 2 or above (50% NYHA 2 in APOLLO3 and 42% NYHA 2 or above in this retrospective study).

Following patisiran treatment, most Belgian patients (*n* = 8) reported stable or improved assessments for most of the neurological and cardiological parameters, with only a few individual cases with worsening statuses (for FIM, and NYHA scores) recorded at the end of the data collection period. These findings are consistent with those reported in the APOLLO Phase 3 trial and to a similar extent are also in line with the HELIOS-A Phase 3 trial assessing the efficacy of vutrisiran in hATTR patients with polyneuropathy [20, 30]. Cardiac involvement was not reported for all patients (both in the retrospective study and in the phase 3 APOLLO study), and no deterioration in LVEF was reported in the patients with cardiac dysfunction.

During the study, one patient discontinued treatment due to heart failure and was probably treated too late in his disease course. This observation reflects the recent recommendations on monitoring symptomatic hATTR amyloidosis patients, indicating that early intervention is key to achieve better patient outcomes [27].

This study has several limitations, such as a small population sample preventing analytic statistical analyses, a limited follow-up period, incomplete data for specific parameters, the lack of Quality-of-Life data and some cardiac parameters (echo, biomarkers, etc.). Due to its retrospective nature, we could not collect all the planned information as not all parameters are routinely collected in standard practice. Prospective data collection initiatives in line with new guidelines described by Adams et al. 2021 [27] can further increase the insights on hATTR amyloidosis patients treated with patisiran in real-world clinical practice.

## Conclusions

Treatment with patisiran in a cohort of adult Belgian patients with hATTR amyloidosis with polyneuropathy, stages 1 and 2, resulted in clinical stabilization or slight improvement of the polyneuropathy with unchanged FAP stage for many of the patients at the last time point compared to baseline, as well as a stabilization of the cardiac function (assessed by NYHA score, ECG and LVEF) in most of the patients. This observation is unlike the expected clinical deterioration seen in natural history control studies of untreated patients with this often-devastating disease. Patient characteristics were akin to those included in the APOLLO trial. Thus, we conclude that the efficacy of patisiran in Belgian real-world clinical practice is generally consistent with results reported in the clinical trials.

## Supplementary Information

Below is the link to the electronic supplementary material.

Supplementary file1 (PDF 433 kb)

## Data Availability

The data that support the findings of this study are not openly available due to the sensitive nature of these data and are available from the corresponding author upon reasonable request.
