# Supplementary material for: A retrospective survey of patients with hereditary transthyretin-mediated (hATTR) amyloidosis treated with patisiran in real-world clinical practice in Belgium
Source: Acta Neurol Belg. 2023 Feb 24;123(3):1029–37. doi: 10.1007/s13760-023-02188-z (PMC10238330; doi:10.1007/s13760-023-02188-z)
Supplement: Supplementary file 1 — Supplementary file1 (PDF 433 kb) [file 13760_2023_2188_MOESM1_ESM.pdf]

Retrospective survey of patients with hereditary transthyretin-mediated amyloidosis treated with Onpattro® (patisiran-LNP), in real-world clinical practice in Belgium

Sponsor's name and address:

Alnylam Belgium B.V.B.A.

Robert Schumanplein street 6

1040 Brussels

Belgium

Date and version of the protocol:

10/FEB/2021 – Version 1.0

TABLE OF CONTENTS

1 SYNOPSIS 4
2 LIST OF ABBREVIATIONS AND DEFINITION OF TERMS 7
3 LIST OF TRADEMARKS 8
4 SURVEY 8
5 BACKGROUND INFORMATION AND SCIENTIFIC RATIONALE 10
5.1 Background 10
5.2 Survey rationale 12
6 OBJECTIVES 12
6.1 Primary objective(s) 12
6.2 Secondary objective(s) 12
7 SURVEY DESIGN 13
8 PATIENT POPULATION 13
8.1 Selection of the sites 13
8.2 Inclusion/exclusion criteria for patients 14
9 DATA MANAGEMENT 14
9.1 Patient identification 14
9.2 Data collection 14
10 ASSESSMENT OF SAFETY 18
11 STATISTICAL ANALYSES 19
11.1 Endpoints 19
11.1.1 Primary Endpoints 19
11.1.2 Secondary endpoints 20
11.2 Cohorts 20
11.3 Planned analyses 20
11.4 Sample size considerations/power calculation 21
12 CONDUCT OF THE SURVEY 21

12.1 Operational procedures ... 21
12.2 Adherence to protocol and amendment process ... 21
12.3 Source documents and access to source data ... 21
12.4 Ethics and protection of human subjects ... 21
12.5 Survey discontinuation ... 22
13 ADMINISTRATIVE PROCEDURES ... 22
13.1 Confidentiality and disclosure of information ... 22
13.2 Reporting and publication plans ... 22
13.3 Recording of data and retention of documents ... 23
14 REFERENCES ... 24

LIST OF TABLES

Table 1 Data collected by timepoint for the Onpattro® subcohort ... 16

List Of Figures

Figure 1 Survey Timelines ... 13

1 SYNOPSIS

Title: Retrospective survey of patients with hereditary transthyretin-mediated amyloidosis (hATTR) treated with Onpattro® (patisiran-LNP) in real-world clinical practice in Belgium.

Short Title: Onpattro® survey

Sponsor: Alnylam Pharmaceuticals

Phase: Phase IV retrospective chart review.

Survey rationale: This retrospective survey will be conducted to collect data needed in the context of the Convention between Alnylam and the Belgian Health Insurance Agency (Rijksinstituut voor Ziekte- en Invaliditeitsverzekering / Institut national d'assurance maladie-invalidité (RIZIV/INAMI) relating to the conditional reimbursement of Onpattro®. The results of this survey will be incorporated in a report to the Belgian Health Insurance Agency and due by June 1st 2021.

The survey aims to gather information about the real-world impact of the reimbursement of Onpattro® on epidemiology, the clinical practice and its associated resource use of adult hATTR patients in Belgium; on the real world usage of Onpattro® and the clinical outcomes associated with its use.

Objectives:

Primary:

- To determine current hATTR patient numbers and clinical practice in terms of treatments used for all Belgian patients included in the total cohort, on 01 Feb 2021.
- To determine the clinical impact of Onpattro® use in adult patients with hATTR, in real world circumstances, in the Onpattro® subcorhort in terms of neurological and cardiac symptoms , for the period starting from 01 July 2018 (start of Expanded Access Program [EAP]), up to 01 February 2021.
- To establish real-world Onpattro® use for adult hATTR patients treated with Onpattro® in the Onpattro®subcohort, for the period starting from 01 July 2018 (start of EAP), up to 01 February 2021

Secondary:

- To determine the clinical impact of Onpattro® use in adult patients with hATTR, in real world circumstances, in the

Onpattro® subcorhort in terms of Quality of Life (QOL), for the period starting from 01 July 2018 (start of EAP), up to 01 February 2021.

Survey Design:

Type of design: observational, retrospective, multi-center, descriptive chart review.

Duration of the survey:

- data collection form completion period: 01 April 2021 to 16 April 2021;
- Onpattro® treatment period covered by the questionnaire: 01 July 2018 to 1 February 2021;
- Data analysis and report: 18 April 2021 to 15 May 2021.

Data Collection: Data collection form completion by investigator (or site personnel).

Endpoints

Primary:

Onpattro® treatment use in terms of:

- Treatment start & stop dates (with reason for stopping)
- Prescribed dosage of Onpattro® (in mg/kg) at the beginning of each cycle
- Total number of Onpattro® vials consumed, at the end of each cycle
- Total number of Onpattro® treatment cycles
- Total number of Onpattro® vials at each timepoint (if available)

Onprattro® treatment compliance in terms of:

- Number of missed Onpattro® treatment cycles per year (if any)
- Treatment interruptions (if any), with dates of treatment discontinuation and treatment reinitiation (with reason for interrupting)

Neurological endpoints:

- FAP score (1, 2) as described by Coutinho (1980)
- EMG status (worsened, stable or improved)
- MRC S-S score (scale of 0-6)
- Functional independence measurements (FIM) score (scale of 1-7)
- NIS score (scale of 0-244)

- ACTIVLIM score (impossible, difficult, easy)
- CADT score (using a scale of 0-4; total normal score of 16 in women and 20 in men)
- VAS score for pain (using a visual analog scale)
- PND score (0, 1, 2, 3a, 3b, 4)

Cardiological endpoints:

- Orthostatic hypotension (yes or no)
- NYHA score (class I-V)
- ECG status (worsened, stable or improved)
- Echocardiography status (worsened, stable or improved) + Cardiac Output percentage

Secondary: QoL endoints

- EQ5-D score
  - EQ5-D type
  - EQ5-D score
- SF-36 or SF-12 score (transformed to 0-100 scale; higher scores indicate better health-related QoL)

Target population: Adult patients with hATTR with polyneuropathy stage 1 or 2.

Site selection and number: 7 sites of NeuroMuscular Reference Centers (NMRC) in Belgium of which 5 sites currently have patients treated with Onpattro®.

Sample size: Approximately 30 patients in the total cohort (meeting the criteria for inclusion), of which approximately 9-12 patients treated with Onpattro®, in the Onpattro® sub-cohort.

Inclusion & Exclusion criteria: Adult patients (≥18 years old) diagnosed with hATTR with polyneuropathy, and confirmed by genetic testing will be included in this survey. There are no specific exclusion criteria.

2 LIST OF ABBREVIATIONS AND DEFINITION OF TERMS

The following abbreviations and special terms are used in this protocol.

Abbreviation	Definition
**BMI**	Body Mass Index
**CATD**	Compound Autonomic Dysfunction Test
**CI**	Confidence Interval
**EAP**	Expanded Access Program
**ECG**	Electrocardiogram
**EMG**	Electromyography
**FAP**	Familial Amyoid Polyneuropathy
**FIM**	Functional Independence Measurements
**hATTR**	Hereditary Transthyretin-Mediated Amyloidosis
**IEC**	Institutional or Independent Ethics Committee
**INAMI**	Institut National d'Assurance Maladie-Invalidité
**IV**	Intravenous or Intravenously
**LNP**	Lipid Nanoparticle
**MRC S-S**	Medical Research Council Sum-Score
**mRNA**	Messenger Ribonucleic Acid
**mBMI**	Modified Body Mass Index
**mNIS**	Modified Neuropathy Impairment Score
**NIS**	Neuropathy Impairment Score
**NYHA**	New York Heart Association
**OLT**	Orthotopic Liver Transplantation
**PND**	Polyneuropathy Disability

Abbreviation	Definition
**QoL**	Quality of Life
**QOL-DN**	Quality of Life – Diabetic Neuropathy
**RIZIV**	Rijksinstituut voor Ziekte- en Invaliditeitsverzekering
**RNA**	Ribonucleic Acid
**RNAi**	Ribonucleic Acid Interference
**TTR**	Transthyretin
**VAS**	Visual Analog Scale

3 LIST OF TRADEMARKS

Onpattro®

4 SURVEY

For questions regarding this protocol, please contact:

Institutions

Sponsor name: Alnylam Pharmaceuticals
Address: Alnylam Belgium B.V.B.A., Robert Schumanplein street 6, 1040 Brussels, Belgium
Contact Person: Sébastien Tilleux
Phone Number: +32 471982973
E-mail:stilleux@alnylam.com

Contributing authors

**Protocol Author:**	Jésabelle Kibanda, Scientific writer, Modis Life Sciences for Alnylam<br>Erol Gabor, Scientific writer, Modis Life Sciences for Alnylam
**Agreed by:**	Sebastien Tilleux, Medical Lead Alnylam Belgium BVBA

5 BACKGROUND INFORMATION AND SCIENTIFIC RATIONALE

5.1 Background

Hereditary transthyretin-mediated amyloidosis (hATTR) is a rare, life-threatening, autosomal dominant multi-systemic disease caused by mutations in the transthyretin (TTR) gene that results in progressive, chronically debilitating morbidity and mortality. The estimated European prevalence of hATTR is 0.14 per 10,000 (between 5000 to 6000 patients), with the majority of cases in Portugal, France, Italy, and the United Kingdom [Patisiran].

There are over 120 reported TTR genetic mutations associated with hATTR, and almost all patients are heterozygous for the mutated TTR allele [Connors, 2003; Falk, 2012; Ando, 2013]. Historically, two clinical syndromes of hATTR have been described: familial amyloidotic polyneuropathy (FAP), and familial amyloidotic cardiomyopathy, both of which are characterized by amyloid deposits comprised of both mutant and wild-type TTR [Yazaki, 2000].

The clinical manifestations of the length-dependent, symmetrical sensorimotor neuropathy are the result of amyloid-mediated injury to large and small peripheral nerve fibers. Sensory abnormalities include painful dysesthesias in the feet and hands, as well as loss of sensation leading to thermal burns involving the feet and hands and to joint injury in the lower limbs. Progressive muscle atrophy and motor weakness in both lower and upper limbs leads to impairment of ambulation and inability to perform other activities of daily living such as holding eating utensils or a drinking glass or managing buttons and zips. Autonomic dysfunction results in debilitating orthostatic hypotension, severe gastrointestinal symptoms (including early satiety, chronic nausea/vomiting, and both diarrhea and constipation), bladder dysfunction with dysuria and urinary retention, as well as cardiac arrhythmias [Connors, 2004; Plante-Bordeneuve, 2011; Ando, 2013]. Cardiac infiltration with amyloid leads to cardiomyopathy characterized by heart failure due to both diastolic and systolic dysfunction, conduction disturbances and arrhythmias [Connors, 2004; Benson, 2007; Plante-Bordeneuve, 2011; Ando, 2013; Swiecicki, 2015]. This constellation of progressive morbidity results in severe disability, wasting due to gastrointestinal malabsorption and malnutrition, and profound loss of quality of life. Death usually results from heart failure or infection, with a median survival of 4.7 years (range 1.3 to 24.8 years) following diagnosis [Plante-Bordeneuve, 2011; Swiecicki, 2015].

In terms of symptoms, four stages of hATTR with polyneuropathy (FAP stages) are distinguished. Patients with stage 0 disease are asymptomatic, patients with stage I (mild) disease are ambulatory, patients with stage II (moderate) disease are ambulatory but require assistance, and patients with stage III (severe) disease are bedridden or wheelchair-bound [Ando, 2013].

Since many of the systemic manifestations of the disease are caused by liver-derived circulating TTR, several treatment approaches aimed at reducing the amount of circulating amyloidogenic protein are currently used for hATTR: orthotopic liver transplantation (OLT), TTR tetramer stabilizers (including tafamidis) and gene silencing by ribonucleic acid interference [RNAi]).

Onpattro® is a synthetic RNAi therapeutic, containing patisiran (ALN-18328), a small interfering RNA targeting TTR messenger RNA (mRNA) formulated as lipid nanoparticles (LNP) to target delivery to hepatocytes in the liver, the primary source of TTR protein in the circulation. The therapeutic hypothesis is that the reduction of liver-derived circulating amyloidogenic TTR protein by Onpattro® will reduce the deposition and promote the stabilization or clearance of TTR amyloid deposits, thereby stabilizing or improving the disease manifestations including polyneuropathy and cardiomyopathy and improving overall health.

Efficacy data were collected in 3 core studies in patients with hATTR with polyneuropathy, including a randomized (2:1 Onpattro® to placebo), double-blind, placebo-controlled Phase 3 study called APOLLO (Study 004), a Phase 2 open-label, single-arm, long-term follow-up extension study (Study 003), and a open-label, single-arm, long-term follow-up extension study for patients completing Study 004 or Study 003 (Study 006). Across all 3 studies, Onpattro® was administered as an approximately 80-min intravenous (IV) infusion at a 300 µg/kg dose every 3 weeks, and all patients were administered premedication (including corticosteroids, H1 and H2 blockers, and paracetamol) to reduce the risk of infusion related reactions.

The pivotal Phase 3 APOLLO study enrolled 225 patients (global 148 on patisiran-LNP and 77 on placebo) [Adams, 2018]. Patients were randomized at 44 sites in 19 countries from North America, Europe, Asia Pacific and Central/South America from December 2013 through January 2016. APOLLO was designed to assess the effect of patisiran-LNP relative to placebo on neuropathy and cardiomyopathy. The primary efficacy endpoint was change from baseline in the mNIS+7 composite neurologic impairment score (NIS) at 18 months. Secondary endpoints included quality of life (Norfolk Quality of Life – Diabetic Neuropathy [QOL-DN] score), measures of motor strength (Neurologic Impairment Score – Weakness), disability (Rasch-built Overall Disability Scale), gait speed (10-meter walk test), nutritional status (calculation of modified body mass index [mBMI]) and autonomic symptoms (Composite Autonomic Symptom Scale-31).

APOLLO met its primary endpoint, modified NIS (mNIS)+7. An improvement in neuropathy compared to baseline (mNIS+7 change of <0 points) at 18 months was seen in 56.1% (95% confidence interval [CI]: 48.1%, 64.1%) of patients on Onpattro® compared to only 3.9% (95% CI: 0.0%, 8.2%) on placebo (odds ratio of 40.0, p=1.82x10^15). The effect of Onpattro® on mNIS+7 was observed across all patient subgroups defined by age, sex, ethnicity, geographic region, TTR genotype, neuropathy severity, disease stage, prior TTR tetramer stabilizer use and cardiac subpopulation. APOLLO also met all of its secondary endpoints, including the key secondary endpoint of Norfolk QOL-DN [Adams, 2018].

Onpattro® received a marketing authorisation in the European Union on 27 August 2018 for the treatment of hATTR. An Expanded Access Program (EAP), was launched on 01 July 2018, to allow patients with hATTR to receive Onpattro®.Onpattro® received a temporary reimbursement from Belgian Health Insurance Agency (Rijksinstituut voor Ziekte- en Invaliditeitsverzekering / Institut national d'assurance maladie-invalidité [RIZIV / INAMI]) on 01 December 2019.

The recommended dose of Onpattro® is 300 µg/kg administered via intravenous infusion once every 3 weeks. Dosing is based on actual body weight. For patients weighing ≥100 kg, the recommended dose is 30 mg. Vitamin A supplementation at approximately 2500 IU vitamin A per day is advised for patients treated with Onpattro®.

5.2 Survey rationale

This retrospective survey will be conducted in order to collect data needed in the context of the Convention between Alnylam and the Belgian Health Insurance Agency (Rijksinstituut voor Ziekte- en Invaliditeitsverzekering / Institut national d'assurance maladie-invalidité [RIZIV / INAMI]) when granting prolongation of the reimbursement of Onpattro®. The results of this survey will be incorporated in a report to the Belgian Health Insurance Agency and due by June 1st 2021.

The survey aims to gather information about the real-world impact of the reimbursement of Onpattro® on epidemiology, the clinical practice and its associated resource use of adult hATTR patients in Belgium; on the real world usage of Onpattro® and the clinical outcomes associated with its use.

6 OBJECTIVES

6.1 Primary objective(s)

- To determine current hATTR patient numbers and clinical practice in terms of treatments used for all Belgian patients included in the total cohort, as on 01 Feb 2021.
- To determine the clinical impact of Onpattro® use in adult patients with hATTR, in real world circumstances, in the Onpattro® subcorhort in terms of neurological and cardiac symptoms, for the period starting from 01 July 2018 (start of EAP), up to 01 February 2021.
- To establish real-world Onpattro® treatment use for adult hATTR patients treated with Onpattro® in the Onpattro®subcohort, for the period starting from 01 July 2018 (start of EAP), up to 01 February 2021.

6.2 Secondary objective(s)

- To determine the clinical impact of Onpattro® use in adult patients with hATTR, in real world circumstances, in the Onpattro® subcorhort in terms of Quality of Life (QOL), for the period starting from 01 July 2018 (start of EAP), up to 01 February 2021

For the endpoints of this survey, refer to Section 11.1.

7 SURVEY DESIGN

Type of design: Retrospective, multi-center, descriptive chart review.

Survey groups: single group without comparator.

Mode of data collection: Data collection form.

Estimated survey period : The questionnaire will cover the Onpattro® treatment period from 01 July 2018 to 1 February 2021. The survey will be performed between 01 April 2021 and 15 May 2021 (data collection between 01 April 2021 and 16 April 2021, and data analysis and report between 18 April 2021 and 15 May 2021).

An overview of the survey timelines is presented in Figure 1.

Figure 1 Survey Timelines

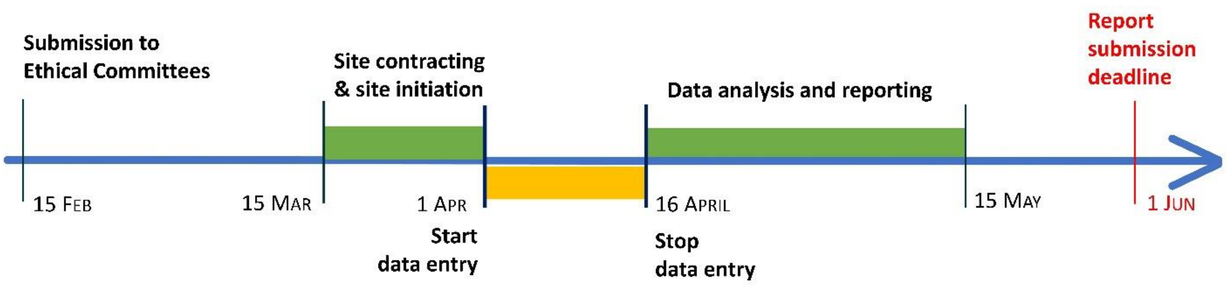

8 PATIENT POPULATION

The population will consist of adult patients (≥18 years old) with hATTR confirmed by genetic testing, with stage 1 or stage 2 polyneuropathy.

8.1 Selection of the sites

Seven sites of NeuroMuscular Reference Centers (NMRC) in Belgium manage hATTR patients. It is estimated that approximately a total of 30 patients will be included from these 7 sites, of which approximately 9-12 patients treated with Onpattro® will be included in the Onpattro® sub-cohort. As 2 of the NMRC sites do not currently have patients treated with Onpattro®, only data for the total cohort will be collected for these sites. In the 5 remaining NMRC sites, data will be collected for the total cohort and the Onpattro® sub-cohort.

The 7 participating centers are as follows:

- Universitair Ziekenhuis (UZ) Leuven,
- Universitair Ziekenhuis Antwerpen (UZA),
- Universitair Ziekenhuis (UZ) Brussel,
- Universitair Ziekenhuis (UZ) Gent,
- Cliniques universtitaires Saint-Luc (CuSL),
- Centre Hospitalier Régional (CHR) de la Citadelle,
- Hôpital Erasme - Cliniques universitaires de Bruxelles.

8.2 Inclusion/exclusion criteria for patients

Inclusion criteria: adult patients (≥18 years old) with a diagnosis of hATTR with polyneuropathy, confirmed by genetic testing.

Exclusion criteria: there are no specific exclusion criteria.

9 DATA MANAGEMENT

9.1 Patient identification

Each patient will be given a unique number to maintain data confidentiality when the patient is included in the survey and is retained as the primary identifier for the patient throughout the survey's duration. Pseudonymized data (anonymous to non-site staff) from patients' medical records will be recorded in the data collection form.

9.2 Data collection

Data recorded in patients' medical records from 01 July 2018 up to 01 February 2021 (observation period) will be collected by site personnel using a data collection form developed by Modis Life Sciences. A data entry assistant from Modis will be available to help record data on the data collection form, upon request by the investigator. Onpattro® treatment data will be obtained from the NMRC site pharmacy.

No personal identifiable information will be collected. After the data entry will be finalized, data will be exported in a format that will allow for data analysis. The sponsor will receive anonymized individual data.

No source-document verification will be performed. Throughout the survey, data management will conduct a verification of completeness of the data and a verification of any inconsistencies in collected data. In case of missing or inconsistent data, queries will be sent to the site for correction.

Data collection for the total cohort: the total number of patients by treatment (liver transplant, tafamidis, Onpattro®) will be collected as aggregated data.

Data collection for the Onpattro® subcohort: data for the Onpattro® subcohort will be collected as described in Table 1.

Table 1 Data collected by timepoint for the Onpattro® subcohort

Parameters collected	Timepoints			
	At EAP treatment initiation[1]	At first reimbursement	At reimbursement prolongation[2]	Last evaluation timepoint[3]
Age	X	X[4]		
Weight	X	X	X	X
mBMI	X	X	X	X
Treatment before Onpattro®	X	X[4]		
Onpattro® start date	X	X[4]		
Prescribed dosage of Onpattro®	X	X	X	X
Number of Onpattro® vials		X[1]	X	X
Onpattro® treatment stop (with date and reason)	X	X	X	X
Onpattro treatment interruption (with date and reason)	X	X	X	X
Onpattro® reinitiation[5]			X	X
Number of cycles		X	X	X
Neurological parameters				
Polyneuropathy and FAP stage[6] (if applicable)	X	X	X	X
NIS score	X	X[5]	X[5]	X[5]
EMG	X	X	X	X
MRC S-S	X	X	X	X
FIM score	X	X	X	X
Activity limitations (measured by ACTIVLIM)[5]	X	X	X	X

Parameters collected	Timepoints			
	At EAP treatment initiation[1]	At first reimbursement	At reimbursement prolongation[2]	Last evaluation timepoint[3]
CADT[5]	X	X	X	X
VAS score for pain[5]	X	X	X	X
Polyneuropathy disability (PND) score[5]	X	X	X	X
Cardiological parameters				
Orthostatic hypotension	X	X	X	X
NYHA score	X	X	X	X
ECG	X	X	X	X
Echocardiography	X	X	X	X
QoL parameters				
EQ5-D[5]	X	X	X	X
SF-36 or SF-12[5]	X	X	X	X

[1] for patients that were included in the EAP
[2] or no longer treated/still on initial reimbursement
[3] up to 01 February 2021, if available
[4] For patients not included in the EAP
[5] if available
[6] as defined by Coutinho (1980)

CADT - Compound Autonomic Dysfunction Test; **ECG** - Electrocardiogram; **EMG** - Electromyography; **FAP** - Familial Amyoid Polyneuropathy; **FIM** - Functional Independence Measurements; **mBMI** - Modified Body Mass Index; **MRC S-S** - Medical Research Council Sum-Score; **NIS** - Neuropathy Impairment Score; **NYHA** - New York Heart Association; **PND** - Polyneuropathy Disability; **QoL** – Quality of Life; **SF-36/12** - ShortForm 36/12; **VAS** - Visual Analog Scale.

10 ASSESSMENT OF SAFETY

No treatments are administered as part of this survey and collection of any treatment-related effects, both safety and efficacy, are out of scope. There will be no specific safety data collection in this survey. However, Investigators must comply with all Adverse Event (as defined below), Other Safety Information (as defined below) and Product Complaint (as defined below) reporting obligations under applicable laws in Belgium.

Investigators must send copies of all reports (including but not limited to interim study reports, final study reports, etc.) submitted to any health authority or regulatory agency regarding an Alnylam product in parallel to Alnylam at: patientsafety@alnylam.com or Fax: 1-289-846-5210.

Investigators shall ensure that any personal information collected about individuals complies with applicable privacy and data protection laws and regulations.

In addition, after receiving the data collection form from physicians, the data will be screened by Modis to identify any potential adverse event (as described below) any Other Safety Information (as defined below) and any Product Complaint (as defined below), which will be reported to Alnylam PV department for further assessment within 24 hours. Alnylam PV department may contact the reporting physician to obtain further information needed to fulfill its reporting obligations.

Definition of an Adverse Event (AE): An AE means any untoward medical occurrence in a patient or Clinical Trial subject administered a Medicinal Product and which does not necessarily have to have a causal relationship with this treatment. An adverse event can therefore be any unfavorable and unintended sign (for example, an abnormal lab finding) symptom, or disease temporally associated with the use of a Medicinal Product, whether or not considered related to this Medicinal Product.

Definition of Other Safety Information: Other Safety Information means circumstances where a report does not include an AE per se, but nevertheless needs to be reported to Alnylam. These circumstances include:

- Use of a product during pregnancy or breastfeeding
- Overdose
- Abuse
- Misuse
- Medication error
- Occupational exposure
- Lack of therapeutic efficacy
- Off-label use
- Suspected transmission via a medicinal product of an infectious agent

- Suspected or confirmed counterfeit/falsified medicinal product

Definition of Product Complaint (PC): Any written, electronic, or oral communication that alleges deficiencies related to the identity, quality, durability, reliability, safety, effectiveness, or performance of a medicinal product, medical device, or combination product after it is released for distribution. Product complaints are typically non-medical in nature; however, it is possible that complaints could be associated with an adverse event.

11 STATISTICAL ANALYSES

11.1 Endpoints

11.1.1 Primary Endpoints

Total cohort:

Number of patients with hATTR

- Number of patients with hATTR by type of treatment

Onpattro Subcohort:

Onpattro® treatment use in terms of:

- Treatment start & stop dates (with reason for stopping)
- Prescribed dosage of Onpattro® (in mg/kg) at the beginning of each cycle
- Total number of Onpattro® vials consumed, at the end of each cycle
- Total number of Onpattro® treatment cycles
- Total number of Onpattro® vials at each timepoint (if available)

Onprattro® treatment compliance in terms of:

- Number of missed Onpattro® treatment cycles per year (if any)
- Treatment interruptions (if any), with dates of treatment discontinuation and treatment reinitiation (with reason for interrupting)

Neurological endpoints:

- FAP score (1, 2) as described by Coutinho (1980)
- EMG status (worsened, stable or improved)
- MRC S-S score (scale of 0-6)
- Functional independence measurements (FIM) score (scale of 1-7)

- NIS score (scale of 0-244)
- ACTIVLIM score (impossible, difficult, easy)
- CADT score (using a scale of 0-4; total normal score of 16 in women and 20 in men)
- VAS score for pain (using a visual analog scale)
- PND score (0, 1, 2, 3a, 3b, 4)

Cardiological endpoints:

- Orthostatic hypotension (yes or no)
- NYHA score (class I-V)
- ECG status (worsened, stable or improved)
- Echocardiography status (worsened, stable or improved) + Cardiac Output percentage

11.1.2 SECONDARY ENDPOINTS

Onpattro Subcohort: the following data will be collected if available

QoL endoints

- EQ5-D score
    - EQ5-D type
    - EQ5-D score
- SF-36 or SF-12 score (transformed to 0-100 scale; higher scores indicate better health-related QoL)

11.2 Cohorts

The total cohort will include all patients meeting the inclusion criteria (i.e. with a diagnosis of hATTR confirmed by genetic testing), for which aggregated data will be collected.

The Onpattro® sub-cohort will include patients with hATTR that have been treated with Onpattro® from the start of the Expanded Access Program (EAP) in 01 July 2018, up to 01 February 2021, for which individualized data will be collected.

11.3 Planned analyses

The analysis of the collected information will only consist of descriptive data such as frequencies, aggregated averages, standard deviations and ranges. No formal statistical analysis plan is set out, neither has there been any statistical hypothesis formulated. The final report of this survey will consist of a descriptive presentation of the collected data.

This is an observational survey and only data collected in routine clinical practice will be used. Only data available at the time of the survey are to be recorded, hence no substitution,

imputation, or other correction methods will be used to complete missing values. Statistical analyses will only be performed on parameters collected in sufficient numbers.

11.4 Sample size considerations/power calculation

No formal power calculation will be performed as no statistics will be performed to compare groups.

12 CONDUCT OF THE SURVEY

12.1 Operational procedures

This survey must be carried out in compliance with the survey description and Alnylam's standard operating procedures and in accordance with any applicable regulatory requirements.

The following activities are outsourced and will be performed by Modis Life Sciences:

- Development of data collection form and survey protocol
- Site contracting
- Independent or Institutional Ethics Committee (IEC) submission
- Site initiation
- Survey management: instructions and follow-up, data cleaning and archiving.
- Survey data analysis and reporting.

The following activities will be performed by the Sponsor:

- Site selection

12.2 Adherence to protocol and amendment process

Any change or addition to this survey protocol requires a written amendment and must be approved by the Sponsor and IEC before the change or addition can be considered effective.

12.3 Ethics and protection of human subjects

As per Belgian law, retrospective studies fall outside clinical trial legislation (07 May 2004). However the protocol and information document for patients will be submitted to the medical ethics committees of the participating centers, for approval. This submission will be carried out by Modis Life Sciences.

For patients that are not included in the Onpattro® subcohort, no written informed consent will be obtained as only aggregated data with no link to the individual patients or medical records can be made.

For patients included in the Onpattro® sub-cohort, patients will be informed in written about the study including:

- The purpose of the study
- Data collected in the study
- Measures put in place to protect their data

Patients will have the opportunity to object to the use of their data in the study. In case the patient objects to the use of their data in the study, their data will not be collected.

12.4 Source documents and access to source data

All source documents will be maintained at the sites. Alnylam Pharmaceuticals will not have access to any source data or documents.

12.5 Survey discontinuation

The Sponsor reserves the right to discontinue the survey at any time. The sites reserve the right to discontinue from survey participation at any time. All data generated up to the time of withdrawal from the survey will be used for the analysis.

13 ADMINISTRATIVE PROCEDURES

13.1 Confidentiality and disclosure of information

Disclosure and confidentiality will be adhered to as defined in the survey agreement executed between Modis Life Sciences and the participating sites.

13.2 Reporting and publication plans

Modis Life Sciences will prepare a survey Report within 1 month after completion of the last data collection form.

In case of publications in biomedical journals, the Sponsor will ensure to follow the guidelines established by the International Committee of Medical Journal Editors and published in its Uniform Requirements of Manuscripts Submitted to Biomedical Journals.

Publication of data subsets from individual institutions participating in multicentre studies should not precede the primary manuscript, and when developed should always reference the primary publication of the entire survey.

13.3 Recording of data and retention of documents

Original documents will be archived as required by the local law.

14 REFERENCES

Adams, D., A. Gonzalez-Duarte, et al. (2018). « Patisiran, an RNAi therapeutic, for hereditary transthyretin amyloidosis.” N Engl J Med 379: 11-21.

Ando, Y., T. Coelho, et al. (2013). "Guideline of transthyretin-related hereditary amyloidosis for clinicians." Orphanet Journal of Rare Diseases 8(31).

Benson, M. and J. Kincaid (2007). "The molecular biology and clinical features of amyloid neuropathy." Muscle Nerve 36(4): 411-423.

Connors, L. H., A. Lim, et al. (2003). "Tabulation of human transthyretin (TTR) variants, 2003." Amyloid 10(3): 160-184

Connors, L., T. Yamashita, et al. (2004). "A rare transthyretin mutation (Asp18Glu) associated with cardiomyopathy." Amyloid 11(1): 61-66.

Coutinho, P., Martins da Silva, A., Lopes Lima, J. and Resende Barbosa, A. (1980) Forty years of experience with type I amyloid neuropathy. Review of 483 cases. In: Glenner, G., Costa, P. and de Freitas, A. (eds), Amyloid and Amyloidosis. Amsterdam: Execerpta Medica, pp. 88–98.

Falk, R. H. (2012). "Senile systemic amyloidosis: are regional differences real or do they reflect different diagnostic suspicion and use of techniques?" Amyloid 19 Suppl 1: 68-70.

Patisiran Orphan Maintenance Assessment Report; EU/3/11/857 - EMA/OD/142/10

Plante-Bordeneuve, V. and G. Said (2011). "Familial amyloid polyneuropathy." Lancet Neurol 10(12): 1086-1097.

Swiecicki, P. L., D. B. Zhen, et al. (2015). "Hereditary ATTR amyloidosis: a single-institution experience with 266 patients." Amyloid 22(2): 123-131.

Yazaki, M., T. Tokuda, et al. (2000). "Cardiac amyloid in patients with familial amyloid polyneuropathy consists of abundant wild-type transthyretin." Biochem Biophys Res Commun 274(3): 702-706.
